# Temporal dynamics and fitness consequences of coalition formation in male primates

**DOI:** 10.1098/rspb.2021.2626

**Published:** 2022-06-08

**Authors:** Christof Neumann, Lars Kulik, Muhammad Agil, Antje Engelhardt, Anja Widdig

**Affiliations:** ^1^ Research Group of Primate Behavioural Ecology, Department of Human Behaviour, Ecology and Culture, Max-Planck-Institute for Evolutionary Anthropology, Leipzig, Germany; ^2^ Research Group of Behavioural Ecology, Institute of Biology, Faculty of Life Sciences, University of Leipzig, Germany; ^3^ Junior Research Group of Primate Sexual Selection, Department of Reproductive Biology, German Primate Center, Göttingen, Germany; ^4^ Courant Research Centre ‘Evolution of Social Behaviour’, Georg-August-Universität, Göttingen, Germany; ^5^ Faculty of Veterinary Medicine, Bogor Agricultural University, Bogor, Indonesia

**Keywords:** cooperation, coalition, dominance, temporal dynamics, reproductive success

## Abstract

Coalition formation is one of the most striking forms of cooperation found in animals. Yet, there is substantial variation between taxa regarding the mechanisms by which coalitions can result in fitness consequences. Here, we investigate the influence of coalitions on dominance rank trajectories and subsequently on reproductive success in wild male crested macaques (*Macaca nigra*) at Tangkoko Nature Reserve (Sulawesi, Indonesia). We observed 128 coalition events involving 28 males and tested how a variety of coalition properties and factors related to the social environment influenced future male rank. We further used genetic paternity analysis of 19 infants conceived during the study to assess male reproductive success. Our results show that males participating in coalitions achieved higher-than-expected future ranks, while coalition targets had lower-than-expected future ranks. Additionally, all-up coalitions had stronger effects on rank than all-down and bridging coalitions, and these were modulated by the relative strength of coalition partners versus targets. Finally, higher ranking males were more likely to sire infants than lower ranking males. These results provide important insights regarding the mechanisms underlying coalition formation and support the idea that one major path by which coalitions can affect fitness is through influencing male dominance trajectories.

## 1. Introduction

The evolution of cooperation is a hallmark of behavioural biology, with coalitions being one of its most impressive manifestations [1,2]. Coalitions are joint aggressive events by at least two individuals (participants or partners) directed towards at least one other individual (target) [1]. Coalitions appear to be limited in their taxonomic distribution, occurring mostly in mammal species, particularly primates, but also in some bird species [3,4].

Classic models on primate coalitions focused mainly on aspects of resource defence among females. In most gregarious primate species, females remain in their natal group and form bonds and support networks, preferentially with kin [5], in order to defend access to food resources [6]. However, male primates usually migrate from their natal group around the time of reaching adulthood [7], which limits the possibilities to interact and form coalitions with relatives after dispersal. Although if male relatives are available, relatedness between males can determine who a male forms coalitions with [8]. Therefore, in the context of female philopatry, the consequences of coalitions among males and the conditions under which they occur are far more puzzling than among females and need to be considered in a different framework because kin selection cannot be readily invoked as the sole explanation ([9], see also [10] for an example of non-kin coalitions in the philopatric sex).

One formal model exists that explains the conditions under which coalitions among primate males should occur and what fitness consequences should be expected (Pandit/van Schaik-model, from here on: PvS model [9,11]). This model distinguishes two mechanisms. First, at least one of the partners increases in dominance rank, therefore benefitting in the long-term (assuming that higher rank translates into higher reproductive success). Second, coalitions among males may level mating distributions by targeting and displacing a male from monopolizing a fertile female so that at least one of the partners gains direct mating access. While coalitions of both types occur in male primates [11], there are only few empirical studies providing evidence for the pathway between coalitions, dominance and reproductive success [12–14].

An important characteristic determining whether coalitions can have rank-changing (or levelling) effects is coalition configuration, i.e. the relative rank of the target with regard to the participants. In all-up coalitions, all participants rank below the target, whereas in the bridging configuration at least one partner ranks above the target while at least one partner ranks below. Both configurations are profitable such that at least one participant benefits in ways that would not be available to him if he were on his own. The key difference is that bridging coalitions are always feasible, i.e. the combined strength of the participants is larger than that of the target because one participant is higher ranking than the target. All-up coalitions, by contrast, do not need to be feasible if two very weak (low-ranking) partners challenge a very strong (high-ranking) target, but can be feasible if two participants of similar strength target an individual of only slightly more strength [15]. Finally, in all-down coalitions, neither partner can directly profit except maintaining the status quo [9]. Here, all participants are already higher ranking than the target and can therefore beat him on their own during contests. Partly because they are always highly feasible by definition, all-down coalitions are considered as not directly beneficial by the PvS model, but may constitute a counter-strategy employed to prevent all-up and bridging coalitions from occurring [5,9].

Despite considerable research effort, we can identify at least three major gaps in our knowledge about coalitions and their consequences. First, coalitions and their consequences are traditionally studied from the perspective of the cooperating individuals, i.e. what are the benefits for participants. Only implicit in this approach are the consequences for targets; whenever somebody rises in rank someone else needs to drop. In order to gain a complete picture, it is important to integrate consequences for participants and targets. Second, existing studies focus either on coalition rates (events per time) or describe coalitions as case studies. There is a clear lack of knowledge regarding the consequences of coalitions from an event perspective, i.e. we do not know what consequences single coalition events have. Third, the question of which time-frame rank-changing coalitions work in has, to our knowledge, never been directly asked. Researchers usually select time units whose relevance in terms of dominance rank dynamics for the species studied are mostly unclear. In the empirical studies mentioned so far, the rank-changing consequences of coalitions were measured over time periods ranging in duration from one month to two years (e.g. [12,13]), though case studies suggest that these effects can also occur within shorter times (e.g. [16]).

Here, we aim to fill these gaps by integrating data from participants and targets, as well as focusing on single coalition events and the temporal consequences related to these events. We study coalitions in crested macaques (*Macaca nigra*), which represent a particularly interesting study system because the adult male hierarchy is highly dynamic with frequent migration events, relatively short alpha tenure and considerable temporal variation in the relationship between rank and reproductive success [17,18].

Our overall goal was to investigate the effects of coalitions on male dominance trajectories and ultimately assess how dominance relates to reproductive success. First, we quantified the effects of coalition characteristics, primarily configuration, feasibility (relative strength) and time elapsed since coalition event, on subsequent dominance status for both participants and targets, separately. Second, we assessed how dominance status of a given male around the date of infant conception predicted the probability of paternity.

## 2. Methods

### (a) Study subjects and data collection

We studied two groups of crested macaques in the Tangkoko Nature Reserve, Sulawesi, Indonesia between March 2009 and May 2011 [17]. Each group comprised up to 85 individuals, with the number of adult males being present at any given time ranging between 7 and 18. All animals of both groups were completely habituated to human observers and adults were individually recognizable.

We collected behavioural data using focal animal sampling [19] of 37 adult males (mean = 66.1 h, range = 0.6–130.0 h per male, total = 2447.2 h). Focal protocols lasted up to 60 min during which continuous data on aggressive behaviour involving the focal male and any group member were recorded. These data were used to calculate rates of coalitionary behaviour. We also recorded coalitions opportunistically, which we combined with the focal data for the main analysis. We defined a coalition as simultaneously occurring aggression by at least two individuals (participants) directed towards at least one male (target) upon which the target left or fled the participants.

### (b) Paternity assessment

We assessed paternity from 19 infants conceived during our study. In brief, we extracted DNA from faecal samples. Genotypes were considered definitive when at least two different samples of the same individual produced the same results in at least four amplifications for heterozygotes and six for homozygotes [20]. We assigned paternity only when exclusion and likelihood calculations revealed the same father [18]. More details can be found in the electronic supplementary material.

### (c) Analysis

In this section, we summarize the design of the models we employed and the variables we used. More details can be found in the electronic supplementary material.

For each event, we assigned each male his role in the coalition, i.e. either as target (victim), or as participant (partner) in aggression directed at a target.

We used Elo-rating to assign each male a score of individual dominance strength (‘dominance rank’, [17,21]). Since we were interested in dominance trajectories, we calculated Elo-ratings multiple times for each male. We started on the day a coalition was observed (‘current rank’, day 0) and used increments of 10 days up to 120 days (future rank). These future ratings served as the response variable in our main analysis.

We assigned coalition events one out of three configurations. Coalitions were considered all-down (all participants had higher Elo-ratings than the target), all-up (all participants had lower Elo-ratings than the target) or bridging (at least one participant had a higher Elo-rating than the target and at least one participant had a lower Elo-rating than the target).

We calculated feasibility as the difference between the sum of Elo-ratings of all participants and the Elo-rating of the target, as measured on the day of the coalition. Prior to this calculation, we standardized ratings such that the highest rating of all males present in the group that day was set to 1 and the lowest to 0. A large feasibility value indicates that the combined ‘strength’ of the participants was greater than the ‘strength’ of the target, whereas small (negative) values indicate that combined strength of participants was smaller than the one of the target.

Since we were interested in the effects of coalition characteristics on future male rank, we organized our dataset in such a way that each coalitionary event is linked to multiple future time points and the targets' and participants' ranks at these time points as response variable. To do so, we re-organized the dataset such that each original coalition event was represented 12 times (once for each time point). In addition to our main predictors of interest (role, configuration, feasibility, days since event and interactions between these terms), we also incorporated a number of control variables (group identity, hierarchy stability index, male competition index, an auto-correlation term, male rank on the day of the coalition, age and aggression rate). The random effects structure accounts for variability in male ID and coalition event ID, as well as a number of random slopes for the main predictors. We built linear mixed effects models with the lme4 package in R v.4.1.1 [22]. More details on the model design and diagnostics can be found in the electronic supplementary material.

We used likelihood ratio tests (LRTs) to assess whether the full model (with all main effects and interactions) was different from an informed null model. This null model contained the same random structure as the full model, but lacked all fixed terms that included any of the main predictors.

To assess the relationship between rank and paternity success, we used a generalized linear mixed model with a binomial error structure. We modelled whether the Elo-rating during the assumed time window around conception predicted the likelihood of siring a given infant for all males present in the group during the time of this infant's estimated conception. We calculated the conception window as the 11-day time window centred around the most likely conception date. We included male ID as a random intercept and controlled for individual male age, and for hierarchy stability and competition index at the group level. Given the dynamics of our study system and the focus on *conception events*, we did not analyse the paternity data in temporarily aggregated time blocks (see electronic supplementary material).

## 3. Results

The mean number of males participating in coalitions was 2.2 (median = 2, range: 2–6). The majority of coalitions was all-down (*n* = 88, 69%), while bridging (*n* = 24, 19%) and all-up (*n* = 16, 13%) coalitions were rarer. More detailed descriptions of coalition configuration and rates can be found in the electronic supplementary material. We did not observe any levelling coalitions where a target male is displaced from monopolizing a fertile female to gain direct mating access.

### (a) Coalition dynamics

The full model investigating variation in future status at different time points was different from the null model (LRT: $x_{22}^{2}=153.75$, *p* < 0.0001, electronic supplementary material, tables S1 and S2 for full results, including estimates for control variables like age and aggression rate, electronic supplementary material, figures S1 and S2). This indicates that the combination of fixed effects that were in the full model (role, configuration, feasibility and time distance) but not in the null model explained significant variation in future status of males after being involved in coalitions.

### (b) Configuration and feasibility

**Figure 1.**
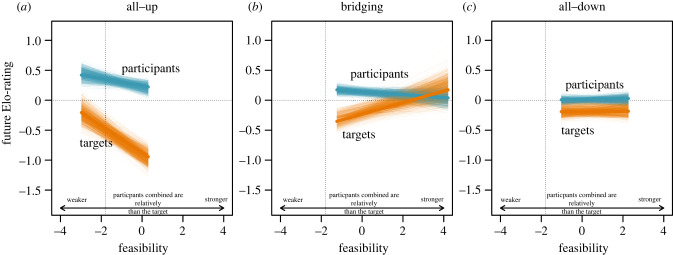
Future status is explained by configuration, feasibility and role. By and large, participants of coalitions had higher future Elo-ratings than coalition targets across all configurations (*a*–*c*). The effect of feasibility was strongest for all-up coalitions (*a*), such that targets of all-up coalitions had relatively lower future ratings if feasibility was large. For bridging (*b*) and all-down (*c*) configurations, both the difference between participants and targets as well as the effect of feasibility was smaller compared to all-up coalitions. The vertical dotted line shows the point where feasibility is zero, i.e. where target strength is equal to the combined strengths of participants. Note that the range along the feasibility axis is restricted to the range of feasibility in the data, i.e. only all-up coalitions have feasibilities that can range into the area left of the dotted vertical line. All-down and bridging coalitions have feasibilities that are constrained to the right side of the vertical dotted line. The thick coloured lines show the model predictions (when all other numerical variables were at their mean, i.e. 0 and categorical predictors were at their reference levels) and the thin coloured lines are model predictions from 1000 bootstraps. (Online version in colour.)

The role of an individual in a coalition, configuration and feasibility were statistically significant predictors of a male's future Elo-rating (LRT, three-way interaction: $x_{2}^{2}=38.29$, *p* < 0.0001, figure 1; electronic supplementary material, table S1). Across all configurations participants generally benefited from coalitions, i.e. participants had higher future Elo-ratings than coalition targets (figure 1). The effect of role, however, differed according to how feasibility interacted with configuration, with differences between targets and participants being most pronounced in all-up coalitions

In all-up coalitions with high feasibility (participants combined were relatively stronger than the target, i.e. lower risk), targets had much lower future Elo-ratings than participants (figure 1*a*). When feasibility was low (participants combined were relatively weaker than the target, i.e. higher risk), the difference between targets and participants was less pronounced. For both participants and targets, future Elo-ratings were smaller when feasibility was high, although this effect was stronger for targets. In other words, for participants, all-up coalitions with low feasibility paid off more than coalitions with high feasibility. For targets, however, low-feasibility coalitions barely affected future ratings, whereas high-feasibility coalitions had drastic negative effects on targets' future ratings.

For bridging coalitions (figure 1*b*), participants benefited most and targets suffered the most (i.e. had the lowest future ratings) from coalitions with low feasibility. With larger feasibility the consequences for both targets and participants converged towards each other, no difference between participants and targets.

For all-down coalitions (figure 1*c*), future Elo-ratings of both participants and targets were largely independent of feasibility, i.e. regardless of feasibility targets had slightly lower future ratings compared to participants.

### (c) Temporal dynamics

**Figure 2.**
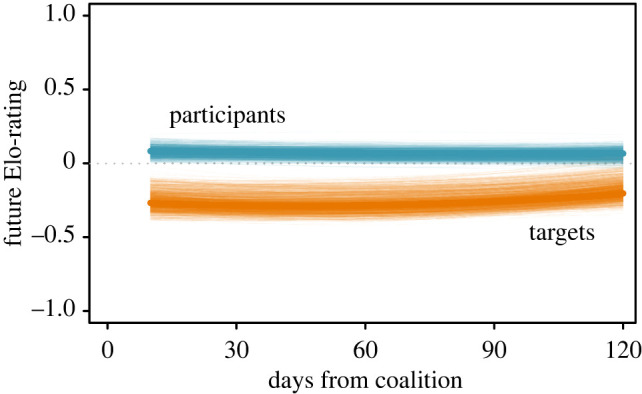
The effect of time with regard to changes in Elo-rating after coalitions for participants and targets. The largest differences between participants and targets occur at small time distances. With increasing time, distance from the date of a coalition event rating changes converge towards zero. Figure shows model predictions (thick lines) and 1000 bootstrapped model predictions (thin lines). (Online version in colour.)

We also found the interaction between role and time distance significant (LRT: $x_{2}^{2}=7.57$, *p* = 0.0227, figure 2). For both targets and participants, our results indicate that coalition consequences are more pronounced shortly after the coalition event (i.e. at small time distances, with the largest difference in future Elo-ratings between participants and targets occurring between days 10 and 30) and appeared to converge to zero with increasing time.

### (d) Reproductive success

**Figure 3.**
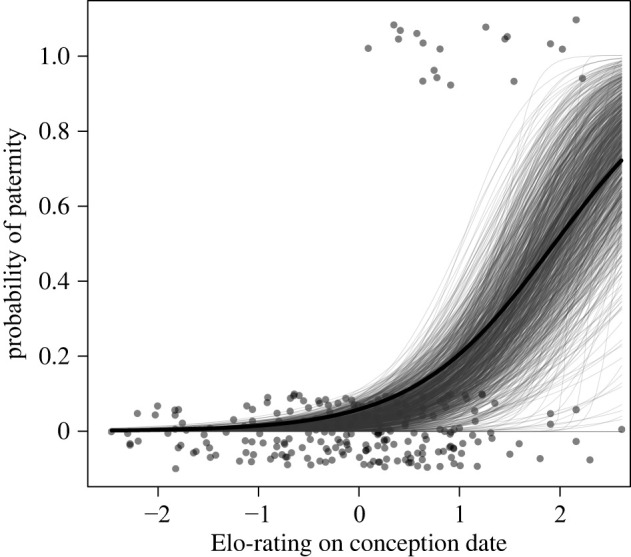
Relationship between Elo-rating and paternity success. Each circle corresponds to a male that was present during the conception of one of the 19 infants in the dataset. Random vertical jitter was added along the *y*-axis to aid in distinguishing data points. Figure shows model prediction (thick line) and 1000 bootstrapped model predictions (thin lines).

Males with higher Elo-ratings during the conception window were more likely to sire infants (*β* = 1.43, s.e. = 0.34, *z* = 4.20, *p* < 0.0001; full versus null model: LRT: $x_{1}^{2}=22.10$, *p* < 0.0001, figure 3; electronic supplementary material, tables S3 and S4).

## 4. Discussion

Our results demonstrate positive relationships between coalitions and dominance rank trajectories, and between dominance status and reproductive success in wild male crested macaques. Coalitions, though relatively rare, appear to be rank-changing and not a mechanism to level mating distributions. The rank-changing consequences are further corroborated by our expected finding that males attained higher ranks after participating in coalitions as compared to when they were targets, although the exact consequences differed between coalition configurations and the degree of feasibility. Given that dominance rank also translates into reproductive success, this suggests that coalition formation among males appears to be one strategy to increase fitness via positive dominance trajectories.

The majority of coalitions was all-down, while all-up and bridging configurations accounted for only about 30% of coalition events. All-down coalitions are theorized as not profitable because they do not provide benefits to coalition partners that either of them could not obtain alone [9]. Rather, all-down coalitions should be the consequence of rank-changing bridging and all-up coalitions to maintain any changes brought about by previous rank-changing coalitions and also as a means to ascertain the reliability of the partners' commitment to each other. Our results suggest an alternative scenario. Changes in rank after all-down coalitions were generally smaller than after all-up and bridging coalitions, for both participants and targets. Beyond pure maintenance of the status quo or providing a buffer against challenges, our results suggest that all-down coalitions can be profitable, at least for some individuals, such that using coalitions males can maintain their rank, which otherwise would drop for reasons other than coalitions, for example due to age trajectories or differences in aggression rates (see electronic supplementary material, figures S1 and S2). Regular all-down coalitions could also be used to induce stress and anxiety in targets and thereby prevent challenges from low-ranking individuals towards high rankers [9,23,24]. It therefore seems that all-down coalitions can be beneficial and profitable, though in a different way from all-up and bridging coalitions and that the non-profitability of all-down coalitions, assumed in the PvS model, needs to be reevaluated.

In addition to the configuration of coalitions, our results indicate that feasibility (relative strength) of coalitions can influence the magnitude and direction of rank changes. The moderating effects of feasibility were most pronounced in all-up coalitions. Here, participants of coalitions with low feasibility, i.e. higher risk, profited the most, while the targets of these coalitions suffered comparatively little, i.e. they had small, if any, decreases in future rank. If feasibility was high, i.e. the coalition had a relatively lower risk from the participants' perspective even though it was aimed at a male higher ranking than each participant, males still had increased future ranks, while targets of these coalitions suffered substantially with dramatically decreased future status. For bridging and all-down coalitions, we found that the moderating effects of feasibility were less pronounced or even appeared to be absent altogether. These results highlight that estimating feasibility of coalitions on a continuous scale is crucial and it is noteworthy that the largest effects of feasibility were observed in the all-up configuration, which is the only configuration in which theoretically the combined strength of participants could be lower than that of the target. Estimating feasibility is not trivial [9,15]. The approach we employed here using standardized Elo-ratings shares a problem with Bissonnette *et al*. [15], who used normalized David's scores. Both have arbitrary scales, i.e. they are bound by group size in the case of David's scores or are constrained between 0 and 1 (our study). This questions the assumption that such scores can be simply added to obtain a valid measure of combined dominance strength for the participants. In their study, Bissonnette *et al*. [15] took advantage of the fact that not all their coalitions were actually successful and used this to estimate a cut-off value of their feasibility measure. If feasibility of a given coalition exceeded this value the coalition was expected to win. Given that in our study all coalitions were won by the participants, we suspect that coalitions among crested macaque males were always feasible in an absolute sense. It is also noteworthy that even though we can set a critical value to evaluate whether or not a coalition is feasible, previous work also showed large variation in this respect: a large proportion of events that were expected to be won because they exceeded the critical feasibility value were in fact lost [15]. Clearly, more research is needed to understand how to estimate best relative coalition strength (feasibility) on a continuous scale.

Furthermore, differences in coalition consequences as a function of feasibility may be influenced by other factors. For example, in the study on male Barbary macaques, coalition targets were more likely to direct counter aggression at their aggressors when feasibility was low as compared to when it was high [15]. Given that feasibility therefore appears to be perceived on a continuous scale (likely with some error [15]), this suggests that the assumption found in current models of coalitions being either feasible or not in a categorical fashion needs to be reevaluated. The study of dyadic conflicts has profited from incorporating such continuous measures of symmetry (e.g. [25]) and future studies will have to investigate whether the degrees of dyadic and polyadic power asymmetries are correlated across species, but also how these properties interact with each other to influence patterns of coalition formation. Likewise, it would also be beneficial that in addition to separating participants from targets, as we did, to analyse the potential consequences of coalitions depending on qualitative differences between participants themselves (like differentiating between high- and low-ranking participants in bridging coalitions, or who of the participants initiated the coalitionary behaviour).

We also found differences between participants and targets with regard to the time course over which coalitions had effects. The most important conclusion here is that the difference in future Elo-ratings between participants and targets occurred at the smallest time points in the future that we modelled, i.e. 10 to 30 days after the coalition. With increasing time distance, Elo-ratings of both participants and targets appear to converge to each other. Furthermore, effects for both participants and targets lack pronounced curvature, suggesting that the effect is in fact linear and hence converging towards 0 with increasing time from the coalition. In other words, the consequences in Elo-rating appear to be most pronounced very shortly after the coalition event. This contrasts somewhat with results from other studies that investigated coalition consequences on much larger time scales up to the magnitude of years [12,13], and it suggests that we need to integrate temporal dynamics in demography (migration rates and residence duration) when deciding on which time scale to investigate coalition consequences.

So far we have shown that coalitions appear to alter dominance trajectories of males in our study population. Given that coalitions did not occur in the context of access to female mating partners, one beneficial means to provide fitness benefits would be if dominance status relates to paternity success. And this is what we found: males with higher Elo-rating at a given conception period were more likely to sire a given infant than males with lower ratings. This relationship between dominance status and paternity success is ubiquitous in a range of animals [26]. Hence, any means to rise in status is likely to improve the chances of an individual to reproduce. Our results show that coalitions may be such a means and it suggests that coalitions can be one viable strategy to improve the dominance status of participants. By the same token, targets of coalitions suffer by decreasing status and this suggests that avoiding being targeted may also prove beneficial.

Dominance trajectories of male primates result from the interplay of a variety of individual properties and are under the influence of environmental and social pressures. Our study describes the consequences of coalitions as one important facet of such a dynamic system. Participating in coalitions provides males with benefits through higher-rank-than-expected without utilization of coalitions and this then translates into higher individual reproductive success. Our study contributes to our understanding of the strategies used by males, to maximize fitness given the different career paths available to them [27] and opens up avenues for important future research into the mechanisms and consequences of coalition formation in primates and other taxa. Interesting remaining questions pertain to partner choice in coalition formation and whether these decisions are made strategically, e.g. via long-term social bonds [13,14], or opportunistically (based on partner availability, which might be unpredictable in the migrating sex due to short and/or non-overlapping tenures). It will also be important to partition the relative contributions of relevant individual features (e.g. age, aggression rates and personality) in shaping dominance trajectories, and how these interact with coalition characteristics.

## Data Availability

Data and code are available from Dryad Digital Repository [28]. The data are provided in the electronic supplementary material [29].
